# Social Isolation, Social Support, and Loneliness Profiles Before and After Spousal Death and the Buffering Role of Financial Resources

**DOI:** 10.1093/geronb/gbac039

**Published:** 2022-04-04

**Authors:** Rosanne Freak-Poli, Claryn S J Kung, Joanne Ryan, Michael A Shields

**Affiliations:** 1 Department of Epidemiology and Preventive Medicine, Monash University, Melbourne, Victoria, Australia; 2 Centre for Health Economics, Monash Business School, Monash University, Melbourne, Victoria, Australia

**Keywords:** Financial resources, Loneliness, Social isolation, Social support, Wealth, Widowhood

## Abstract

**Objectives:**

We provide new evidence on the profiles of social isolation, social support, and loneliness before and after spousal death for older widows. We also examine the moderating effects of gender and financial resources on changes in social health before and after widowhood.

**Methods:**

We use 19 waves of data from the Household, Income and Labour Dynamics in Australia Survey, including 749 widowed individuals and a comparison group of around 8,000 married individuals. We apply coarsened exact matching weights and control for age and time trends. Local polynomial smoothed plots show the profiles of social health from 3 years pre- to 3 years postspousal death. All analyses were stratified by gender.

**Results:**

Spousal death was strongly associated with increased loneliness for women and men, but also an increase in interactions with friends and family not living with the bereaved. For men, financial resources (both income and asset wealth) provided some protection against loneliness. Spousal death was not associated with changes in social support or participation in community activities.

**Discussion:**

We demonstrate that loneliness is a greater challenge of widowhood than social isolation or a lack of social support. Our findings suggest that interventions focusing only on increasing social interactions are unlikely to alleviate loneliness following spousal death. Moreover, policies that reduce the cost of formal social participation may have limited effectiveness in tackling loneliness, particularly for women. Alternative strategies, such as helping the bereaved form a new sense of identity and screening for loneliness around widowhood by health care workers, could be beneficial.

Poor social health is increasingly being recognized as a major concern globally (World Health Organization, 2021), affecting strongly on health and well-being (Gerst-Emerson & Jayawardhana, 2015; Holt-Lunstad et al., 2017). Social health can be broadly defined as relating to “someone’s abilities to adapt in social situations and form satisfying meaningful relationships, and how someone interacts with and is supported by other people, institutions and services” (Freak-Poli et al., 2021, p. 1). Social health is often measured through the related but distinct constructs of social isolation, social support, and loneliness. Social isolation is the lack of social relationships or infrequent social contact with others and can be objectively measured (National Academies of Sciences & Medicine, 2020). In contrast, social support is measured subjectively and is the actual or perceived availability of resources from others, which can include emotional support and/or access to resources (finances, goods, services, or information; Fiorillo & Sabatini, 2015; National Academies of Sciences & Medicine, 2020; Valtorta et al., 2016; Wang et al., 2017). Loneliness is a subjective negative feeling of being isolated (National Academies of Sciences & Medicine, 2020) that has been described as the discrepancy in what an individual perceives as their current situation, relative to their desired combination of the quantity and quality of their social interactions (Peplau & Perlman, 1982; Victor & Yang, 2012). Loneliness is a distressing and pervasive experience (Matthews et al., 2019), that is thus not just about the availability or frequency of social interactions; it is very much a measure of perceived scarcity or having less than you feel you need (Mullainathan & Shafir, 2013).

A large literature has established links between poor social health and diminished immunity, poor diet, sleeplessness, physical inactivity, reduced physical functioning, mental health conditions including depression, reduced cognition and an increased risk of dementia, and ultimately a higher risk of mortality (Cacioppo & Cacioppo, 2014; Cacioppo & Hawkley, 2009; Gerst-Emerson & Jayawardhana, 2015; Holt-Lunstad et al., 2010, 2015, 2017; Liu et al., 2020; Spreng et al., 2020; Szabó et al., 2020; Wilson et al., 2007). In fact, loneliness has been found to increase mortality risk to at least the same extent as obesity and smoking (Flegal et al., 2013; Holt-Lunstad et al., 2010, 2015). Consequently, poor social health is associated with increased health care utilization and therefore large costs to health care systems (Freak-Poli et al., 2022; Kung et al., 2021; Mihalopoulos et al., 2020). Moreover, as might be expected, poor social health is strongly socioeconomically graded, being more prevalent in low socioeconomic status groups (Kahlbaugh et al., 2011; Kung et al., 2021).

However, it is important to distinguish in any analysis between social isolation, social support, and loneliness (National Academies of Sciences & Medicine, 2020), because they have been found to have differential pathways to poor health and disease (Cacioppo & Hawkley, 2003; Menec et al., 2019; Shankar et al., 2011; Smith & Victor, 2019; Steptoe et al., 2013). In particular, social isolation has been shown to have significant associations with mortality and poor health outcomes, even after accounting for loneliness (Ge et al., 2017; Hakulinen et al., 2018; Newall & Menec, 2017; Shankar et al. 2011; Steptoe et al. 2013). Importantly, relationships between indicators of social health are modest, with correlations between loneliness and social support of 0.31, and between loneliness and social isolation of only 0.09 (using Australian data, Kung et al., 2021).

## Widowhood and Social Health

It is inevitable that many individuals will experience the distress of spousal or partner bereavement, that is, the loss of the person who is often their closest confidant, main source of support, and best friend. For some, spousal death comes quickly and unexpectedly, but for others, spousal health may decline over many months or years. Spousal loss can lead to a substantive change in the social health of the bereaved, which can start to occur in the period before death, particularly for those with caregiving duties.

Widowhood is associated with both lower social support and higher loneliness (van der Houwen et al., 2010). People who are widowed are 5.2 times more likely to feel lonely often, compared with people who were in a relationship (Age UK: Love Later Life, 2018). Recent widowhood holds the greatest risk (Savikko et al., 2005), where the odds of becoming lonely may increase by up to 193% (Yang, 2021). A recent study (Lim-Soh, 2021) observed that four aspects of social isolation were constant prior to spousal loss and improved greatly after spousal loss. Contacting and meeting a child was greatest around the time of spousal loss, while meeting friends and attending group activities continued to steadily increase. As for social support, there can be a decline before spousal loss, especially among older adults where longer periods of poor health are common, followed by an increase postloss from family and friends (Utz et al., 2002).

There are several theories relevant to how spousal loss may influence social health. Consistent with theories of social network substitution and compensation (Rook & Charles, 2017; Zettel & Rook, 2004), maintaining or increasing social contact could be a strategy to cope with widowhood. Additionally, according to the theory of socioemotional selectivity, older adults maintain stability in social network and support by selectively focusing on fewer but more intimate relationships to meet their emotional needs (Carstensen, 1992), which interestingly implies both an improvement in social support metrics, and a worsening in social isolation metrics, following widowhood. The stress-buffering theory is the notion that social support can provide stress-buffering effects and thereby reduce loneliness and improve well-being (Cohen & Wills, 1985). Therefore, maintaining or increasing social support could be beneficial for bereavement-related loneliness. A systematic review assessing the relationship between widowhood and health among older adults reported that “… older widows and widowers managed their emotional pain and suffering thanks to support described as social bonds, family support or support from friends …” (Holm et al., 2019, p. 606). Closely aligned with the stress-buffering theory is the social integration perspective, which suggests that social interaction can benefit psychological adjustment to the death of a spouse and overall well-being during widowhood (Berkman et al., 2000). However, research has demonstrated that “social relationships acquired prior to widowhood, or those available in early stages of widowhood, do not appear to explain individual differences in adaptation to loss” (Anusic & Lucas, 2014). Therefore, we conceive that stress-buffering theory is likely more relevant to long-term widowhood, rather than the initial period of widowhood. Perhaps the lack of benefit from social engagement in the early stages of widowhood can be explained by attachment theory, which is the notion that supportive friends cannot compensate the loss of an attachment figure (Stroebe et al., 2007). Therefore, loneliness due to spousal loss may be unaffected by maintaining or improving social connections.

Lim et al.’s (2020) recent conceptual framework is particularly effective at illustrating how social health is linked to major life events, such as spousal loss. The model outlines major life events (termed triggers), risk factors, and correlates of loneliness, spanning demography, health, and socioenvironmental factors. Major life events are not necessarily negative, such as downsizing or relocation, but they may involve a change in social identity that is often combined with social adjustment. Major life events can occur simultaneously, for example, widowhood and financial strain. Additionally, major life events may not have a sudden onset, for example, if a widowed person has savings to cushion the loss of a second pension or deteriorating health that becomes worse over time.

Importantly, people experience major life events differently and they may not directly lead to poor social health. In particular, anticipatory and adaptation effects may differ. Anticipation is a state of expectation, either positive or negative, about an upcoming event or situation. For example, if the spouse has declining health prior to death, an anticipatory effect may be felt preceding spousal death. Adaptation is the ability to adapt to a new situation. For example, a widowed person’s ability to adapt to the death of their spouse may depend on the circumstances surrounding spousal death, and the accompaniment of other major life events. All of these may influence trajectories of grief over time, leading to differential changes in social health metrics.

## Effect Modifiers

As highlighted by Lim et al.’s (2020) conceptual model, there are factors that can influence the effect of major life events. Gender and financial circumstances may be two important modifiers of the impact of widowhood on social health.

### Gender

The sociohistorical context of gender likely modifies how social health is interpreted, the types of social participation, and how spousal loss is experienced. In many societies, women were more likely to undertake unpaid caregiving roles for children, husbands, and parents, and to be social connectors, for example, in organizing gatherings with family and friends. This sociohistorical view has undoubtably affected gender differences in social health expectations and behaviors. Prior research suggests that women are more likely to be active members in community activities (i.e., less socially isolated) and have larger social networks (i.e., be more socially supported) than men (Hu et al., 2021; National Academies of Sciences & Medicine, 2020). However, there are conflicting findings regarding loneliness, with some studies reporting higher rates among men, and others reporting no gender difference (Borys & Perlman, 1985; Hu et al., 2021; National Academies of Sciences & Medicine, 2020). Differential reporting of loneliness by gender, with women more likely to self-label as feeling lonely, could be due to the negative consequences or stigmatization of being lonely being greater for men (Borys & Perlman, 1985).

The sociohistorical context has also influenced marriage, as men tend to pair with younger women in heterosexual relationships. For example, in 2019 in Australia, men were 2 years older than women on average when married, which has been unchanged over the past two decades (Australian Bureau of Statistics (ABS), 2021b). In addition, women tend to live longer than men, meaning that men who die are more often married compared to women who die (52% vs 26% in a 2019 Australian survey; ABS, 2021a). These factors contribute to a greater number of widowed women than men, who spend approximately a decade in widowhood without a partner (Freak-Poli et al., 2017; Karraker et al., 2011).

Considering these facts, spousal loss is likely to be experienced differently between men and women. Studies have suggested that the experience of spousal loss is worse among men than among women, with one reason being that men tend to have a stronger reliance on their spouses as confidants, and for the maintenance of social contacts (Stroebe et al., 2001; Wörn et al., 2020). Another study has suggested that women are more likely to adjust to widowhood than men, as men tend to have greater feelings of loneliness (Carr et al., 2018). Additionally, men and the very old tend to have less social support during widowhood (Isherwood et al., 2012). Furthermore, as women are more likely to undertake social activities with friends and families outside of the home (including membership of different types of social and community clubs), it likely incurs a higher financial cost to participate. We might therefore expect the buffering “effect” of greater financial resources (in particular income) to be higher for social participation. Therefore, gender may be a modifier of the social health during widowhood.

### Financial Circumstances

The availability of economic resources can be considered a key facilitator of lower social isolation and greater social support in widowhood (Arling, 1976; Isherwood et al., 2012). For example, savings may cushion spousal loss as it gives the widowed person the ability to stay in their existing home or time to consider downsizing. Furthermore, savings may prevent or delay the need to return to work after losing a second pension. Additionally, changes to financial and residential circumstances following spousal death may affect social health: A reduced income could mean a lack of ability to purchase and participate in many social activities and a reluctance to undertake social activities due to perceived stigma. Additionally, the bereaved may have left or reduced their participation in the workforce to care for their spouse, which may limit financial resources to socialize before or after spousal death. Similarly, for financial and practical reasons, surviving spouses may no longer be able to (or choose not to) remain in the family home: They may move closer to remaining family, or into residential aged accommodation, which can affect their social health. For others, the alleviation of caring duties following spousal death can allow for reconnection of existing social relationships or the pursuit of new ones. Another pathway, particularly for working-age spouses, is a return to the labor market following bereavement, either out of financial necessity or by choice, which could lead to new social interactions and networks. Furthermore, as discussed earlier, women are more likely than men to socialize outside of the home, potentially incurring a higher financial cost to participate in these activities. Hence, the buffering “effect” of greater financial resources (in particular income) might be gender-specific.

## This Present Study

Bereavement-related loneliness has received relatively little attention (Szabó et al., 2020). From mainly cross-sectional studies, we know that widowhood is associated with worse social health, compared with nonwidowed people. However, people’s circumstances and experiences of life events differ. Hence, it is important to assess change in social health during spousal loss within the same person. To date, no previous study has documented how the trajectories of social isolation, social support, and loneliness, simultaneously, could be differentially affected by widowhood.

In this present study, we examine 6-year trajectories of social health leading up to and following the bereaved after spousal loss (Supplementary Figure 1). We utilize a large longitudinal, nationally representative sample of Australians and rearrange the panel data around the spousal loss event, assessing time as continuous. We allow for anticipatory and adaptation effects, focusing on the period from 3 years pre- to 3 years postspousal loss.

To gain a fuller picture of social health, we examine the trajectories separately for social isolation, social support, and loneliness. Additionally, we assess whether the different components of social health influence each other—that is, we examine whether people with lower social isolation and greater social support experience lower levels of loneliness after spousal loss.

Modeling our understanding of the literature specific to social health during widowhood, we restrict social isolation to contact with persons other than those living with them (Lim-Soh, 2021). Furthermore, we are interested in the type of social contact; therefore, we separate social isolation into informal gatherings with friends/relatives and formal group activities. Based on the discussed theories and conceptual frameworks, we hypothesize that prior to spousal loss, social isolation and social support will worsen. However, after spousal loss, social isolation from friends/relatives and social support will improve, while there will be a greater delay for an improvement in isolation from community activities. Loneliness, on the other hand, will be unchanged prior to spousal loss and worsen greatly after spousal loss. Loneliness may plateau or decrease toward the end of the observation period.

To shed some light on potential interventions, we will also investigate whether gender plays a modifying role and whether financial resources can protect or buffer against deteriorations in social health following spousal death. People in later life are likely to have financial resources invested in their home and have low disposable income (colloquially termed “asset rich, cash poor”). Hence, we examine both wealth and income separately.

## Method

### Data

We analyzed data from the Household, Income and Labour Dynamics in Australia (HILDA, 2001–2019), an annual survey tracking the economic and subjective well-being, and labor market and family dynamics, of a large sample of Australian households. The survey began in 2001 with a nationally representative sample of 7,682 Australian households occupying private dwellings, where members providing at least one interview were followed in subsequent waves. In 2011, 2,153 households were added to the sample, to retain its cross-sectional representativeness. The sample also gradually extends each year to include new household members when original household compositions change. Data from HILDA have already been used to shed light on the health and well-being trajectories following spousal death (Anusic & Lucas, 2014; Frijters et al., 2011; Kettlewell et al., 2020; Kung, 2020; Szabó et al., 2020), although there has not been a focus on the different dimensions of social health. For the present study, we retained only individuals with nonmissing social health information and nonmissing date of spousal death. For individuals with missingness on wealth and household income data, we applied the imputed values provided by the data custodians (Summerfield et al., 2020).

### Measuring Spousal Death

We identified spousal death using two sources of information. Deaths of HILDA sample members have been either matched to official administrative data from the National Death Index, informed by fieldwork interviews, or informed by both sources. There were 2,295 deaths among all 43,770 individuals enumerated at any HILDA wave between 2001 and 2019. Of these, 1,196 deceased individuals could be matched to an enumerated partner who was interviewed in at least one wave. We did not use 447 of these deaths: 180 deaths occurred after their partner had already been recorded as deceased, and 267 deaths occurred to their surviving partner at an age younger than 55 or older than 85. The age restriction was based on the financial effect modifier, as Australians can typically start to access their superannuation at age 55. Therefore, the impacts of spousal death on social health for younger (<55) people will likely be different, for example, due to different labor market attachments, earlier life wealth and income, as well as repartnering behavior. A separate analysis was not possible as there were too few observations of death among people aged younger than 55, especially for widowed men. Similarly, older (>85) people will likely be differentially affected due to wealth, as well as health. This left 749 deaths, of which 59% were informed by both administrative and fieldwork data, 36% fieldwork only, and 5% administrative data only. Our widowed sample thus comprised 550 women and 199 men, and the average age of the widowed person at spousal death was 71.7 years (*SD* = 8.4) for women and 72.8 years (*SD* = 8.2) for men.

Nonwidowed individuals used for comparison were those who, throughout the observation period, have been linked to only one unique partner and never reported being separated, divorced, widowed, or single.

We further matched the two samples on their year of birth via a coarsened exact matching procedure, as have been conducted and reported elsewhere (Kung, 2020). In essence, this procedure creates a stratum for every year of birth: Each individual enters a stratum and is given a weight, where widowed (treated) individuals are weighted one, and control (nontreated) individuals are weighted by the size and composition of their stratum. Strata without at least one widowed and one nonwidowed individual are given zero weight and are effectively excluded from the analysis. The resulting nonwidowed sample comprised 3,816 women and 4,069 men, each with a weight from the matching procedure to be entered in subsequent analysis.

Being widowed and duration since widowhood were defined using the information on the date of interview (and hence assessment of social health) and spousal death. The former variable indicated 1 if the interview occurred in the month of, or months after, spousal death (0 if the interview occurred in the months prior to spousal death, or if the individual was in the nonwidowed sample). As we conceptualize that the relationship between time before/after spousal death and social health is not linear, we categorized time (yearly) for a more accurate depiction of changes in these outcomes over widowhood. To calculate duration since widowhood, we collapsed the months to form indicator variables for each year of widowhood: the reference group was 3 years prespousal death and before (interview occurring ≥25 months before spousal death), 2 years predeath (13–24 months before), 1 year predeath (1–12 months before), 1 year postdeath (0–12 months after spousal death, with 0 reflecting that the interview took place in the month of spousal death), 2 years postdeath (13–24 months after), and 3 years’ postdeath and after (≥25 months after). Nonwidowed individuals took on the value 0 on all six indicators for year of widowhood.

Of the widowed sample comprising 550 women and 199 men, the average number of time points in which they were observed is 4.5 (ranging between 1 and 6), with only 275 women and 101 men having been observed at all six different time points. Given the 19-year time span of the data set, individuals could theoretically have 19 observations. Individuals were uniquely allocated to either the widowed or nonwidowed sample. When individuals had more than one observation in the categories “3 years and before” (prewidowhood) or “3 years and after” (postwidowhood), the observations were averaged.

### Measures of Social Health

Loneliness and lack of social support were measured using “I often feel very lonely” and “I don’t have anyone that I can confide in,” which participants rated on Likert agreement scales from 1 (*strongly disagree*) to 7 (*strongly agree*). Social isolation was measured using “In general, about how often do you get together socially with friends or relatives not living with you?”, rated on a frequency scale from 1 (*every day*) to 7 (*less often than once every 3 months*). This measure of social isolation was chosen to exclude interaction with the bereaved spouse, which would obviously reduce after spousal death. However, a limitation of this measure is that it does not separate spouse from other resident family or friends, possibly overestimating social isolation. For these three outcomes, we used dichotomized versions: For loneliness and lack of support, 1 indicated ratings 5–7, or agreement with the corresponding statement, and 0 for ratings from 1 to 4. For social isolation, 1 indicated reports of less than weekly get-togethers with nonhousehold members, and 0 indicated higher frequencies. Finally, club or association membership was a yes (1) or no (0) response to the question “Are you currently an active member of a sporting, hobby or community-based club or association?”.

### Moderating Variables

Binary gender (men and women) and financial circumstance were assessed as possible effect modifiers. We first used total household wealth—that is, the real value of all financial (e.g., government transfers and pensions, lifetime savings from earnings and stock investments) and nonfinancial assets (e.g., homeownership), minus total debts—which is an important measure of financial resources available to older individuals (Vespa, 2012). Second, we looked at real disposable household income, equivalized using the modified Organization for Economic Cooperation and Development (OECD) equivalence scale (OECD, no date). Using equivalized income essentially adjusts for the different financial resource needs of different household types (size and age composition). The modified OECD scale allocates 1 point to the first adult in the household, 0.5 to each additional person who is 15 years and older, and 0.3 to each child younger than 15 years. Equivalized household income is then calculated by dividing household income by the sum of these points.

We split financial circumstance information (wealth or income) into terciles, at every year of measurement. This was done separately for each gender. To capture prespousal death financial circumstances, we used the tercile value observed closest in time to the 2 years prior to spousal death, for widowed individuals. For nonwidowed individuals, we used the tercile value closest to age 70 (i.e., average age at spousal death minus 2 years).

### Statistical Analyses

We started by using nonparametric kernel-weighted local polynomial plots to document the continuous trajectories for social health outcome measures in the period from 3 years before to 3 years after spousal death. We showed equivalent trajectories for individuals who have been continuously married, after matching them to the widowed sample by (a) year of birth and (b) year of birth and prewidowhood household wealth terciles (Utz et al., 2002). We then used individual fixed-effects regression models to estimate how social health changes with widowhood status and duration (Kung, 2020; Wörn et al., 2020). Such models essentially rely on within-individual (rather than between-individual) differences in the outcome of interest, thus eliminating confounding by observed or unobserved time-invariant individual differences, including scale reporting heterogeneity and traits. As a starting point, we regressed each outcome on binary widowhood status, along with age, age-squared, and year dummy variables as controls, which removes aging and period effects associated with social health. In our preferred specification, we regressed each outcome on the set of indicator variables for year of widowhood, omitting as the reference variable 3 years prespousal death and before, and including the same age, age-squared, and year controls. Estimates on these year of widowhood variables, therefore, reflected differences in the social health outcome between the relevant year of widowhood and the average across observations prior to 2 years prespousal death. All regressions were conducted separately by gender.

To examine the impact of moderating variables, we conducted these regressions separately by predeath wealth or income terciles and plotted the resulting estimates in graphs. Finally, in an extension to this analysis, we included two time-varying measures of financial situation, namely, reporting having experienced a “major worsening in financial situation” in the past year, and the continuous logarithm of equivalized household income. Our intention here was to examine whether the financial resources immediately available to the widowed individual, which may be affected by the event of spousal death, might moderate the relationship between wealth and social health in widowhood. However, we remained cautious in interpreting these findings, given these time-varying measures were potentially endogenous to our outcome.

## Results

Widowed participants were more likely to live to be older, unemployed, have lower education, and net household wealth, compared to the nonwidowed matched sample (Table 1).

**Table 1.** Summary Statistics for Widowed and Nonwidowed Samples

	(1) Widowed		(2) Nonwidowed		p		(3) Nonwidowed (weighted)	
	M	F	M	F	M	F	M	F
N	231	567	4,192	4,226			3,985	4,174
2001 covariates								
Age (mean + *SD* years)	65.8 + 10.6	64.3 + 10.5	53.5 + 11.1	54.2 + 12.3	<.001	<.001	64.4 + 11.4	63.7 + 11.7
Education								
University degree	8.7%	7.1%	16.0%	11.8%	<.001	<.001	8.8%	6.8%
Diploma/certificate	36.8%	16.2%	44.4%	24.0%	.020	<.001	36.7%	16.1%
High school graduate	54.5%	76.8%	39.7%	64.2%	<.001	<.001	54.5%	77.1%
Employed (vs not employed)	29.0%	19.2%	64.5%	48.4%	<.001	<.001	29.0%	18.8%
Net household wealth^a^ (mean + *SD* AU$’000)	606 *+* 666	701 + 972	875 + 1,306	863 + 2,515	<.001	.007	729 *+* 1,216	687 + 1,339

*Notes:* Missing values imputed as SEIFA decile average (using Index of Economic Resources). SEIFA = Socioeconomic Indexes for Areas, which rank regions in Australia on social and economic well-being. Index of Relative Advantage and Disadvantage is used.

^a^Wealth = total assets minus debts.

### Descriptive Results

**Figure 1.**
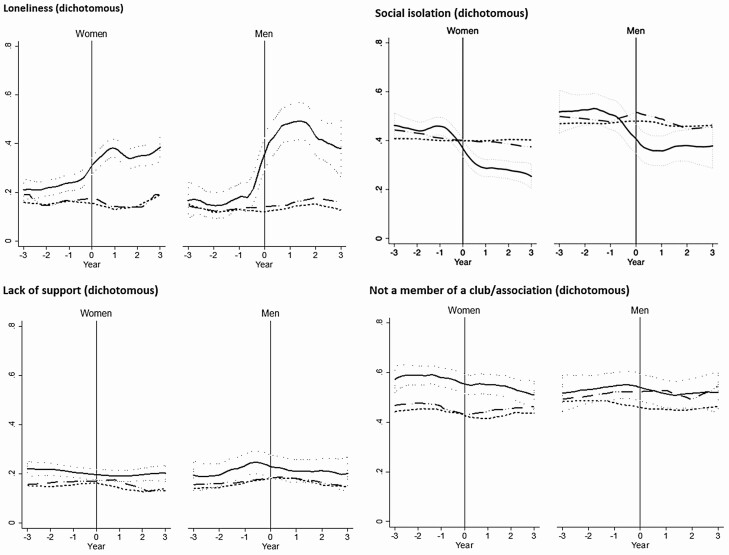
Trajectories of social health, 749 widowed people compared to a matched nonwidowed sample. Trajectories of social health by year of widowhood, using nonparametric smoothed local polynomial plots. Values are coded such that higher values on the *y*-axes reflect poorer social health. Scores are ratings on a 7-point Likert agreement scale. The solid plot represents the widowed sample; the dotted plot represents continuously married controls, matched by year of birth; the dashed plot represents continuously married controls, matched by year of birth and wealth tercile. The shaded area represents 95% confidence intervals. The vertical line at 0 indicates the time of partner death for the widowed sample (women *n*: 231, men *n*: 567), or the average age at which spousal death occurred in the matched nonwidowed sample. Note that “member of club/association” is already binary and no raw score/no further breakdown of response is possible.

We focused on social health trajectories using the dichotomized outcomes (Figure 1) but also showed similar profiles using the raw ratings (Supplementary Figure 2). Three years prior to spousal death, 15%–20% of participants reported feeling lonely. By the time of spousal death, the prevalence of loneliness increased to around 30% for women and 40% for men and continued to increase to around 40% for women and 50% for men approximately a year into widowhood. Those who remained continuously married without spousal loss have relatively constant levels over time, regardless of the matching weights applied (age only, or age and prewidowhood wealth tercile). Even after 3 years, loneliness among the widowed remained elevated and did not drop back to the constant lower level observed among individuals who remained continuously married.

Social isolation followed a very different trajectory. Prior to widowhood, levels were around 40% for women and 50% for men, with little difference seen between soon-to-be widowed individuals and their age-matched counterparts. However, there was a clear decrease in levels of isolation among those experiencing spousal death to around 30% for women and 40% among men, which was not observed among those who remained married without spousal loss. Therefore, postspousal death, widowed individuals were likely to socialize more with nonhousehold members.

There was little change in levels of social support and participation in social activities (captured by club or association membership) throughout widowhood. Widowed women reported on average lower levels of social participation, compared with age-matched, or age- and wealth-matched, women who remained continuously married.

### Fixed-Effects Regression Models

Results for the four dimensions of social health are given in Table 2 (see Supplementary Table 1 when using raw scores). Being widowed predicted a rise in loneliness and fall in social isolation, but not changes in social support and participation. More specifically, Panel 1 shows for women, being widowed predicted a 11.8-percentage-point increase in the likelihood of agreement with “I often feel very lonely,” which corresponds to around a proportionate increase by 68% (i.e., relative to the sample mean). The estimated increase in loneliness for widowed men was around 155%. The drop in social isolation predicted by widowhood corresponded to a proportionate decline by 45% for women and by 31% for men.

**Table 2.** Effects of Widowhood on Social Health From Individual Fixed-Effects Regression Models

	Lonely (I often feel very lonely)		Social isolation (Less than weekly social get-togethers with nonhousehold members)		Lack of social support (I don’t have anyone that I can confide in)		No formal social participation (Not a member of a club or association)	
	Women	Men	Women	Men	Women	Men	Women	Men
(1) Widowhood status								
Widowed	0.118*** [0.084, 0.152]	0.221*** [0.160, 0.281]	−0.185*** [−0.222, −0.147]	−0.151*** [−0.213, −0.089]	−0.014 [−0.042, 0.014]	0.011 [−0.033, 0.054]	−0.017 [−0.056, 0.022]	0.007 [−0.055, 0.069]
(2) Year of widowhood								
−2 years	0.028 [−0.014, 0.070]	−0.015 [−0.078, 0.049]	−0.015 [−0.060, 0.031]	0.012 [−0.065, 0.090]	−0.004 [−0.043, 0.036]	0.017 [−0.047, 0.081]	0.024 [−0.016, 0.065]	−0.001 [−0.068, 0.069]
−1 year	0.025 [−0.018, 0.069]	0.031 [−0.045, 0.106]	0.007 [−0.041, 0.055]	−0.057 [−0.147, 0.033]	0.025 [−0.017, 0.067]	0.080** [0.006, 0.154]	0.027 [−0.017, 0.070]	−0.004 [−0.076, 0.069]
+1 year	0.188*** [0.137, 0.240]	0.310*** [0.222, 0.399]	−0.170*** [−0.217, −0.124]	−0.193*** [−0.274, −0.111]	−0.015 [−0.055, 0.024]	0.033 [−0.035, 0.101]	0.020 [−0.027, 0.066]	0.001 [−0.072, 0.075]
+2 years	0.135*** [0.083, 0.186]	0.373*** [0.280, 0.467]	−0.187*** [−0.238, −0.137]	−0.137*** [−0.226, −0.047]	0.005 [−0.039, 0.049]	0.037 [−0.036, 0.110]	0.003 [−0.048, 0.054]	−0.006 [−0.086, 0.074]
+3 years and after	0.098*** [0.057, 0.138]	0.158*** [0.089, 0.227]	−0.196*** [−0.245, −0.147]	−0.161*** [−0.240, −0.079]	−0.013 [−0.047, 0.022]	0.023 [−0.033, 0.078]	−0.022 [−0.074, 0.030]	0.009 [−0.075, 0.093]
Sample mean	0.172	0.143	0.411	0.482	0.158	0.171	0.506	0.511
Widowed (Obs; Indiv)	6,518; 543	2337; 198	6470; 545	2332; 198	6498; 543	2341; 198	6564; 545	2365; 198
Married (Obs; Indiv)	38,724; 3,871	39,212; 4,095	38,824; 3,873	38,755; 4,107	38,848; 3,871	39,126; 4,098	38,910; 3,875	39,178; 4,110

*Notes:* Obs = number of observations; Indiv = number of individuals. Figures are unstandardized *B* coefficient estimates and 95% confidence intervals (in square brackets). Dichotomized versions of the social health outcomes were used, so estimates can be interpreted as changes in the likelihood of experiencing poor social health. All regressions included age, age-squared, and year dummy variables. For widowed individuals in the Panel (2) regression models, the reference period was over 2 years before spousal death. Interviews are conducted annually, and each individual may have more than one observation in the categories “3 years and before” (prewidowhood) or “3 years and after” (postwidowhood), in which case the observations were averaged.

*p < .10, **p < .05, ***p < .01.

Mirroring the trajectories in Figure 1, Panel 2 shows the difference in social health between the reference period (i.e., over 2 years before spousal death) and the relevant year of widowhood, thereby revealing the trajectory associated with widowhood, after accounting for aging and period effects. For women, average loneliness was around 18.8 percentage points higher over the first year of widowhood compared with the reference period, that is, a proportionate increase of 109%. From the third year of widowhood, the estimated difference was halved to 57%, but remained significant. The trajectory of loneliness for widowed men was about twice the intensity: Average loneliness in the year of spousal death was 31.0 percentage points higher than in the reference period (a proportionate rise by 217%), halving to a 15.8-percentage-point difference (110%) after the second year of widowhood.

Compared with the reference period, average social isolation declined proportionately by around 41% for women and 40% for men in the year of spousal death, compared with the reference period. This difference from the reference period was still at a similar magnitude even after 2 years of widowhood. Consistent with Figure 1, Table 2 presents no changes in social support or participation that can be associated with widowhood status or duration since widowhood.

### Role of Financial Resources

**Figure 2.**
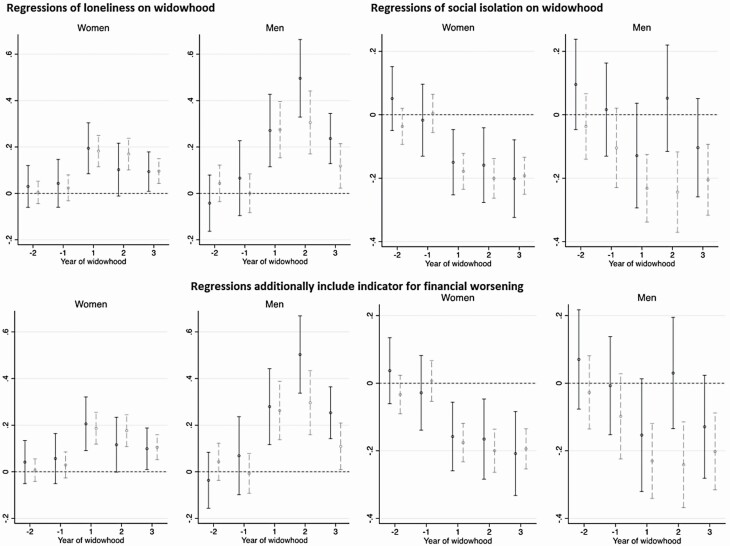
Regressions of loneliness and social isolation on widowhood, and the effect modifier of wealth. Coefficient estimates for year of widowhood, from fixed-effect regressions, conducted separately by wealth groups. Higher values on the *y*-axes reflect deteriorations in social health. The black point estimates are from the regression for the low-wealth group, with the black solid vertical range representing 95% confidence intervals (widowed sample sizes are 134 women, 51 men). The gray point estimates are for the high-wealth group, with the gray dashed vertical range representing 95% confidence intervals (widowed sample sizes are 288 women, 95 men).

**Figure 3.**
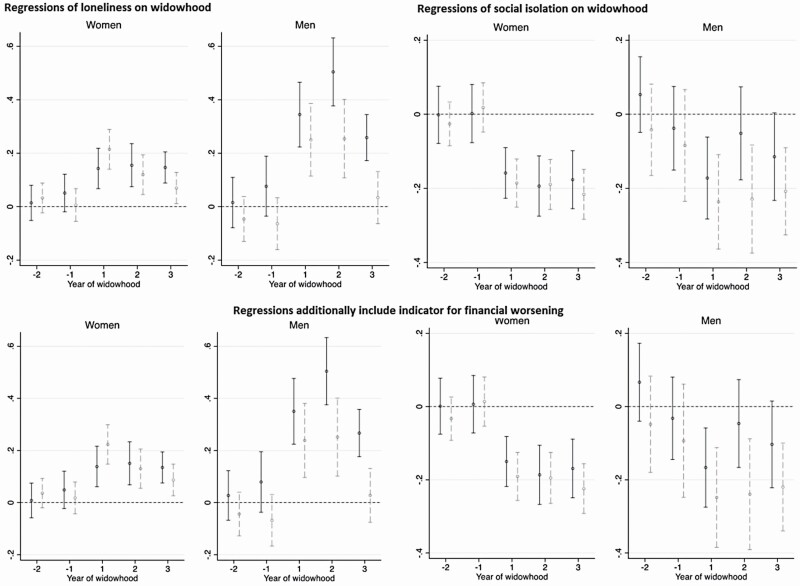
Regressions of loneliness and social isolation on widowhood, and the effect modifier of household income. Coefficient estimates for year of widowhood, from fixed-effect regressions, conducted separately by equivalized household income groups. Higher values on the *y*-axes reflect deteriorations in social health. The black point estimates are from the regression for the low-income group, with the black solid vertical range representing 95% confidence intervals (widowed sample sizes are 220 women, 87 men). The gray point estimates are for the high-income group, with the gray dashed vertical range representing 95% confidence intervals (widowed sample sizes are 261 women, 81 men).

Figures 2 and 3 illustrate the roles of wealth and income in moderating the changes in loneliness and social isolation across time; we left out social support and participation from this analysis, given average levels across widowhood did not deviate from levels over 2 years before spousal death. The first row of Figure 2 presents estimates from conducting the regressions in Panel 2 of Table 2 separately by wealth terciles (measured around 2 years prior to spousal death). For women, there was very little difference in the pattern of increase in loneliness between the wealth groups considered. For men, from the second year of widowhood, the increase in loneliness (compared with the reference period) was larger among those in the bottom wealth tercile, than those in the top two terciles. However, the standard errors were large due to the small sample, and differences in these estimates were not statistically significant.

The social isolation trajectory for women was also similar between the wealth groups. The decline in social isolation after spousal death was greater among higher-wealth men than lower-wealth men; moreover, it was only among the wealthier group that the decline is statistically different from zero. Thus, postspousal death, wealthier men were more likely to engage in weekly (or more) social get-togethers with nonhousehold members.

We then controlled for time-varying measures of available financial resources in these regressions. The second row of Figure 2 plots the resulting estimates after including an indicator for experiencing a major worsening in financial circumstances, whereas Supplementary Figure 3 shows the estimates after including both this indicator and equivalized household income. These patterns did not differ substantively from the first row, implying that the buffering role of wealth—particularly in the case of increased social visits among men—was not modified by the perceived availability of financial resources and the amount available.

In Figure 3, we reestimated the loneliness and social isolation regressions, separately by groups of equivalized net household income, in place of wealth. Similar to wealth, for women, income had virtually no role in moderating the changes in loneliness and social isolation. Among men, while the role of wealth was clearer for social isolation than for loneliness, income moderated both outcomes: higher-income men showed smaller increases in loneliness after spousal death. Moreover, after the second year of widowhood, while higher-income men reverted to average levels measured during the reference period, lower-income men still reported significantly elevated loneliness. Income differences in the social isolation trajectory for men were similar to (albeit slightly less pronounced than) the wealth differences in Figure 2, where the decline in social isolation after spousal death was greater among higher-income men than lower-income men.

We also examined, descriptively, whether the moderating role of income may be explained by perceptions of the availability of financial resources in widowhood. When we included the time-varying indicator for experiencing a major worsening in financial circumstances in the regressions (second row of Figure 3), the pattern of estimates appeared very similar to that without this variable (first row of Figure 3).

## Discussion

Among 749 bereaved women and men, we observed a substantive increase in loneliness and decline in social isolation (contact with friends and family outside the home), directly after experiencing spousal death. However, there were no clear changes in perceived social support or participation in formal social activities before or after spousal death. In contrast, our control sample of around 8,000 individuals who remained continuously married without spousal loss, matched by age and wealth to the widowed sample, reported stable levels on all social health outcomes throughout the period of interest. More specifically, compared with over 2 years before spousal death, in the first year of widowhood, the estimated impact was a proportionate rise in the prevalence of loneliness by around two-fold for women and three-fold for men, after controlling for aging and period effects. There was a gradual decline thereafter, but even after 2 years of widowhood, loneliness remained elevated, where the proportionate rise was around 50% for women and 100% for men, compared with the reference period. The decline in social isolation after spousal death appeared lasting for both women and men, where the proportionate decline in social isolation was around 40% from the reference period. Better social support and lower social isolation did not protect against the increased loneliness associated with widowhood.

We are the first to assess social isolation, social support, and loneliness trajectories both prior to and after spousal loss. We have identified only two studies that have assessed social isolation, social support, and loneliness, where all recruited people were already widowed, preventing within-person comparisons before and after spousal loss (Utz et al., 2014; van der Houwen et al., 2010). We additionally identified seven studies that have assessed social health (either social isolation, social support, and/or loneliness) fluctuations before widowhood, only two of which compared findings to nonwidowed individuals (Isherwood et al., 2012; Lim-Soh, 2021; Utz et al., 2002). Both Isherwood et al. (2012) and Utz et al. (2002) reported greater social support among widowed individuals, relative to nonwidowed individuals. The five remaining studies assessed social health fluctuations among a widowed sample only; two were interested in social support as a mediator or predictor of resilience trajectories (Anusic & Lucas, 2014; Infurna & Luthar, 2017); one assessed social support, loneliness, and depressive symptom trajectory profiles over 12 years before and after spousal loss (Szabó et al., 2019); one reported that veteran widows retained their social support, compared to nonveteran widows who had declines in social support after spousal death (King et al., 2020); and one observed that four aspects of social isolation were constant prior to spousal loss and improved greatly after spousal loss (Lim-Soh, 2021). Furthermore, one of the relevant studies incorporated a loneliness question among the social support measure (Anusic & Lucas, 2014), and only one assessed more than one social health measure independently (Szabó et al., 2019).

Our findings align with past studies on the increase in loneliness following spousal loss (Szabó et al., 2020; Yang, 2021) and the decline thereafter (Utz et al., 2014). Similar to our findings, Szabó et al. (2019) reported increases in loneliness years prior to spousal loss for 62% of participants. The authors also reported less fluctuations in social support, with the majority of participants (70%) having high social support both before and after spousal loss. For 20% of the participants, social support declined and remained chronically low with a small increase in social support after spousal loss for 10% of participants. However, their estimated profiles are not directly comparable with our findings. We found no evidence for changes in the availability of a confidant throughout widowhood, contrary to Ha (2008) who found, using data on around 200 widowed individuals, a significant association between spousal death and loss of a confidant. However, the same study and others (Scott et al., 2007; Utz et al., 2002) also demonstrated increased support from children, friends, and relatives, at different time points postwidowhood, suggesting the ways in which widowed individuals utilize their support network may change as they adapt to widowhood. There may have been a temporary rise in social support which leveled off as the reality of widowhood sets in (Ha, 2008; Utz et al., 2014), or a reorganization of social lives to cope with spousal bereavement (Rook & Charles, 2017). Notably, these mixed findings may be due to differences in perceived support measurement (e.g., emotional vs tangible support, different sources of support).

Our finding that social isolation (with family/friends) starts to improve just before spousal death confirms two studies. Isherwood et al. (2012) reported the average social isolation trajectory started to improve at 5 years prior to spousal loss. The authors also reported that the number of phone contacts with children was greater for widowed people, which may account for some of the improvement also observed in our study (Isherwood et al., 2012). Lim-Soh (2021) observed that four aspects of social isolation were constant prior to spousal loss and improved greatly after spousal loss. In a manner similar to our study, meeting with friends was found to steadily increase after spousal loss until the end of the observation period. However, the author also observed an increase in attending a group after spousal loss, which we did not. We add to these prior findings by demonstrating that social isolation, as well as social support and loneliness, is relatively stable for comparable nonwidowed people. Prior studies have reported that among widowed people, both loneliness and social support seem to naturally decline over the first few years postloss (Powers et al., 2014; Utz et al., 2014). In contrast, we observed that loneliness started to decline a year prior to spousal loss, and that social support was stable over the 6-year observation period.

### Effect Modifiers

Despite the sociohistorical context of gender and social roles, our overall findings did not differ by gender. However, the change in loneliness was greater for men than for women, adding to the literature that “men suffer more” from spousal bereavement (Stroebe et al., 2001). Furthermore, our moderating analysis suggests that only men receive the benefit of prewidowhood wealth and income. In particular, the increase in loneliness after spousal death was smaller for higher-income men than for lower-income men, and the decline in social isolation (with friends/family) was significant only among wealthier men. These are consistent with past work showing that after spousal death, increased contact with children is greater among higher-income individuals (Isherwood et al., 2012), and that the decrease in the likelihood of having a confidant is larger among those experiencing contemporaneous financial problems (Ha, 2008). Our analysis by gender, social health outcomes, and availability of financial resources (an important consideration for older adults, among whom most resources can be in the form of wealth rather than income; Vespa, 2012) further contributes to this small body of evidence.

### Implications

Our findings that after spousal death, there is an increase in informal social get-togethers, but not formal social participation or availability of a confidant, are consistent with the theories of social network substitution and compensation (Rook & Charles, 2017; Zettel & Rook, 2004). In particular, increasing social interactions with friends and relatives may be an active coping strategy for spousal death, but formal social activities (e.g., clubs, organizations) may not be sufficient to provide the emotional support required (Utz et al., 2002). Future empirical research could test whether increased informal interactions indeed compensate for lost emotional ties after spousal death—thereby rendering, as our study showed, little change in the availability of a confidant before and after spousal death.

Moreover, we found that loneliness rises substantially, despite increased social interactions, which offers some support for attachment theory. The emotional and social needs met by these increased interactions are not likely to be sufficient to buffer the stress experienced in the transition to widowhood, especially for men. However, with men, we found that increases in loneliness and social interactions after spousal death are, to an extent, moderated by wealth and income, where those with greater resources show better outcomes. Lower resources may limit opportunities to purchase and participate in meaningful (i.e., stress-buffering) social activities, thereby necessitating care and interventions for men in lower socioeconomic groups. However, our findings may also have implications for money as a coping strategy: In an extension to the stress-buffering theory, Zhou and Gao (2008) discussed the role of money, and how men can be more inclined than women to use the money to deal with pain.

### Limitations

As with many longitudinal studies, our findings are subject to selection effects. Widowed individuals in our working sample (550 women, 199 men) did not necessarily have information on each of the 6 years of widowhood indicators. It is nevertheless reassuring that when restricting the sample to only those observed in all 6 years (275 women, 101 men), our main conclusions were unchanged (Supplementary Figure 3 and Supplementary Table 2). Second, we only had data on the cause of spousal death for just under half of our surviving widowed sample; future research using different data sets may exploit such information to examine whether social health responses differ by the extent to which spousal death was anticipated. Next, the period length selected to analyze social health pre- and postspousal death is annually, to allow for sufficient observations in each period analyzed (particularly for men). Shorter intervals, with a sufficient sample size within each cell, may be more suitable to capture changes in bereavement. Social health measured more frequently using larger samples may shed light on further fluctuations in the trajectory across widowhood (e.g., changes in the first and second quarter after spousal death; Utz et al., 2002).

We were unable to distinguish between social activities undertaken with the person living with the bereaved (i.e., spouse, adult children) for social isolation. Prior social health and widowhood literature has excluded activities with the bereaved’s spouse, but not other people living with them (Lim-Soh, 2021). Therefore, we may be overestimating social isolation in this present study. We were also unable to distinguish between different social network members—friends, children, other relatives—when measuring get-togethers and support. The extent and type of support these members provide could vary, and further quantitative and qualitative evidence may help to improve intervention design.

While we focus on gender and financial circumstances as potential modifiers of the impact of widowhood on social health, it is important to recognize that there are other life events that could be considered as modifiers. For example, later life commonly includes retirement or redundancy, relocation, declining health, life-threatening illness, and/or death of a close friend. All of these events could have modifying effects on the impact of widowhood on social health and could be considered in future research.

Finally, the interpretation of social health constructs may differ across the life course, generations, and cultures. How often we socialize, with whom, and the types of activities that are acceptable can differ. The people around us, from childhood to adulthood, can influence our social health expectations and needs, as well as providing or hindering opportunities to socialize and make connections with people.

As this study was performed using Australian data, the findings should be interpreted within a western view of widowhood, grief, and social health. Australia has well-developed social welfare systems and relatively low social inequality (OECD, 2011). Hence, family and close friends may play a less prominent role in providing welfare and care, and results may not be generalizable to countries with less developed social welfare systems or greater social inequality.

## Conclusion

Bereavement following the death of a spouse is a natural protective process, and the bereaved may choose to withdraw from social interactions or maintain or expand them as part of the process. However, given the strong links between poor social health and health and mortality outcomes, it is of societal interest to ensure that any worsening in social health due to bereavement is not prolonged or intensified. Loneliness seems to be the primary problem following spousal death; we observed no decline in social support or participation and instead an increase in contacts with family and friends. Widowed men suffered more than women, specifically a three-fold compared to a two-fold increase in loneliness after spousal loss. Financial resources were protective for men: Higher incomes were associated with less loneliness, and wealth was associated with more social activities with family and friends. These findings suggest that interventions to increase opportunities for social interaction and participation may not be the most effective way to tackle loneliness. Interventions targeting loneliness directly and tailored to suit the needs of the bereaved individual, such as supported socialization, cognitive behavioral therapy (Fakoya et al., 2020), and social prescribing (Drinkwater et al., 2019; Roland et al., 2020), could be avenues worth pursuing.

## Supplementary Material

gbac039_suppl_Supplementary_Material
