# Supplementary material for: Social Isolation, Social Support, and Loneliness Profiles Before and After Spousal Death and the Buffering Role of Financial Resources
Source: J Gerontol B Psychol Sci Soc Sci. 2022 Apr 4;77(5):956–71. doi: 10.1093/geronb/gbac039 (PMC9071412; doi:10.1093/geronb/gbac039)
Supplement: gbac039_suppl_Supplementary_Material [file gbac039_suppl_supplementary_material.pdf]

Supplementary Material

Figure S1: Conceptual figure of this present study

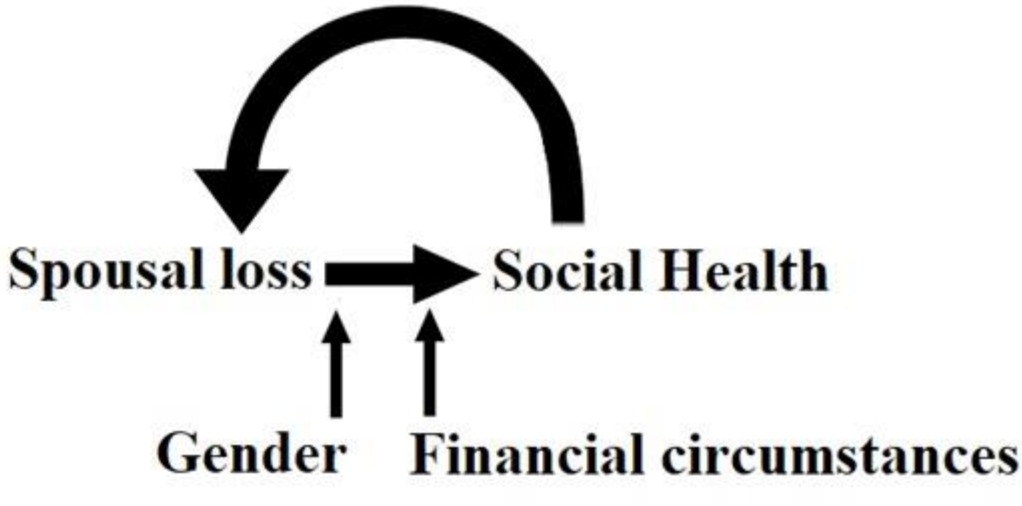

Figure S2. Trajectories of raw social health, 749 widowed people compared to a matched non-widowed sample

Corresponds to Figure 2. Trajectories of social health by year of widowhood, using non-parametric smoothed local polynomial plots. Values are coded such that higher values on the y-axes reflect poorer social health. Scores are ratings on a seven-point Likert agreement scale. The solid plot represents the widowed sample; the dotted plot represents continuously married controls, matched by year of birth; the dashed plot represents continuously married controls, matched by year of birth and wealth tercile. The shaded area represents 95% confidence intervals. The vertical line at 0 indicates time of partner death for the widowed sample (women n: 231, men n: 567), or the average age at which spousal death occurred in the matched non-widowed sample. Note that 'member of club/association' is already binary and no raw score / no further breakdown of response is possible.

Loneliness (raw score)

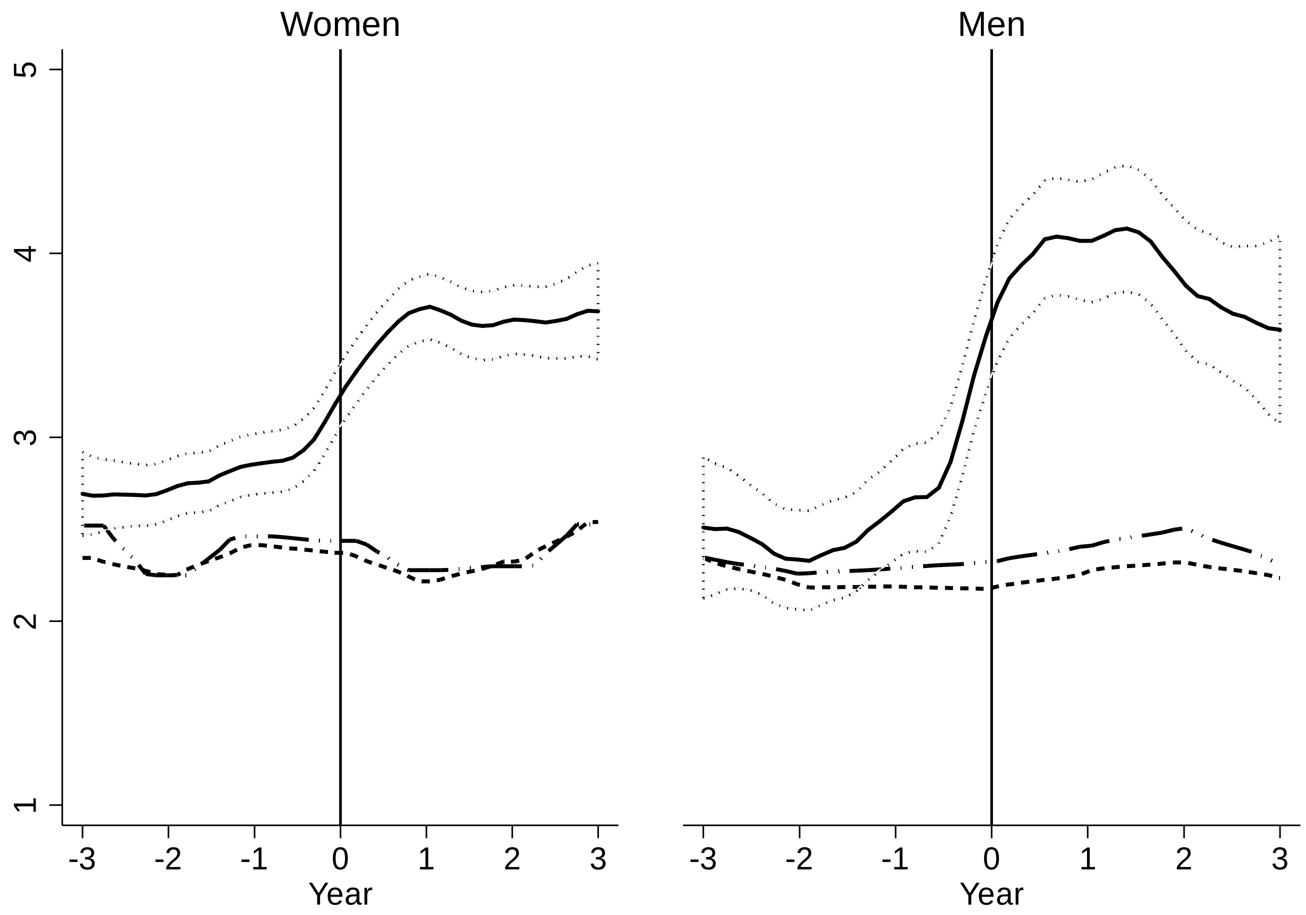

Social isolation (raw score)

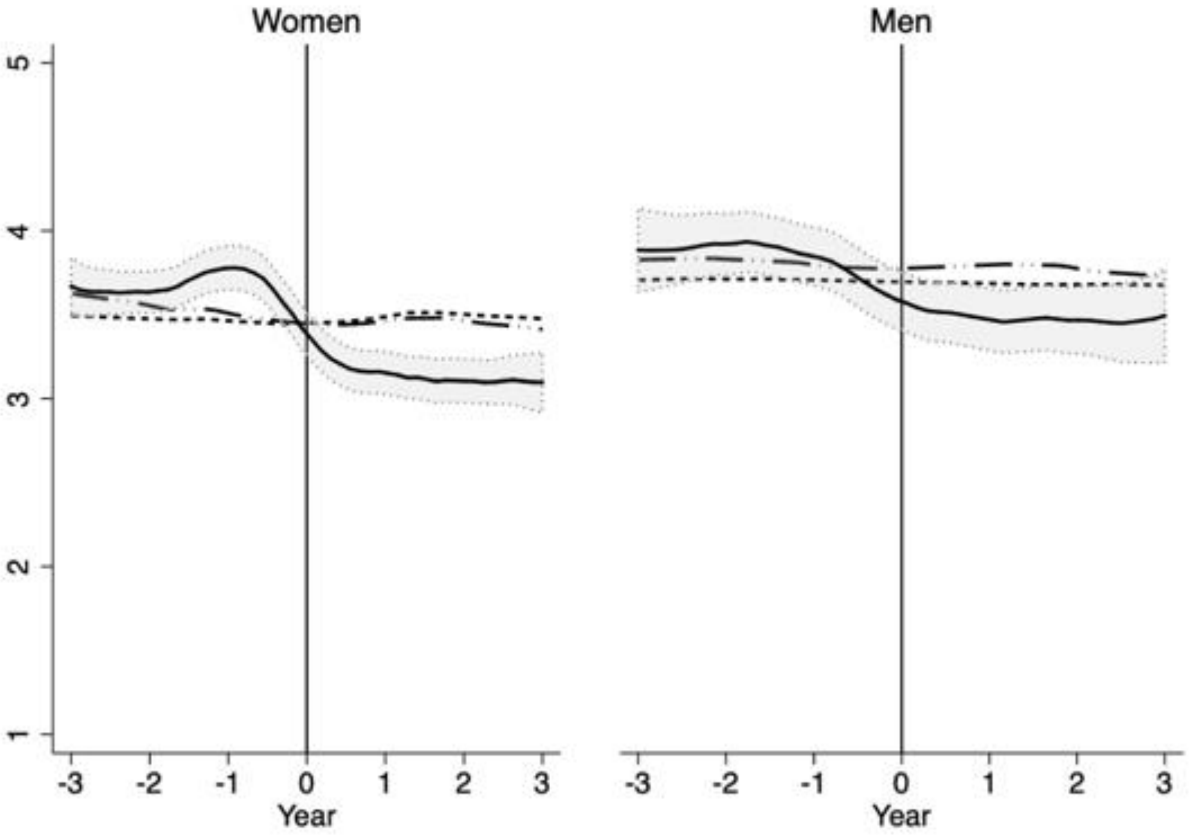

Lack of support (raw score)

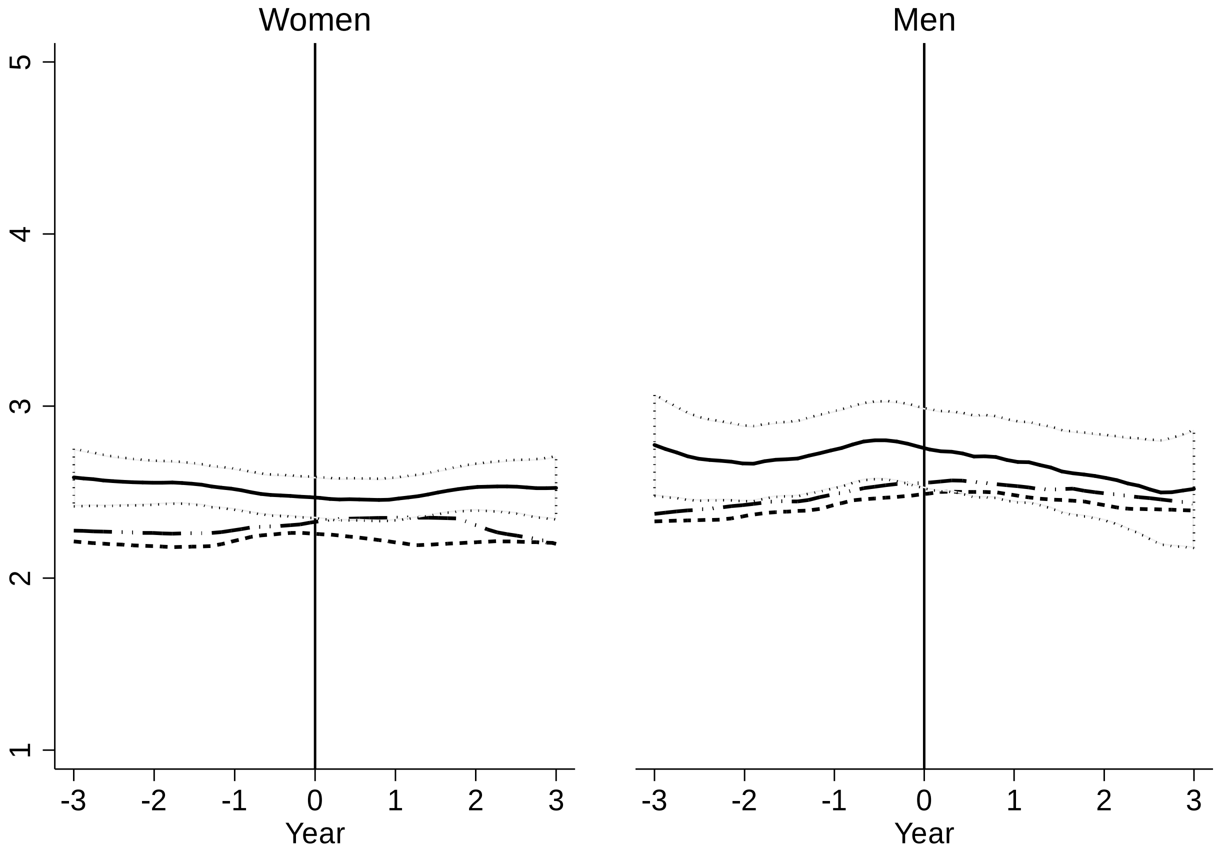

Figure S3. Regressions of loneliness and social isolation on widowhood, and the effect modifier of wealth with additionally including an indicator for financial worsening and log equivalised household income

Corresponds to Figure 2. Coefficient estimates for year of widowhood, from fixed-effect regressions conducted separately by wealth groups. Higher values on the y-axes reflect deteriorations in social health. The black point estimates are from the regression for the low-wealth group, with the black solid vertical range representing 95% confidence intervals (widowed sample sizes are 134 women, 51 men). The grey point estimates are for the high-wealth group, with the grey dashed vertical range representing 95% confidence intervals (widowed sample sizes are 288 women, 95 men).

Loneliness

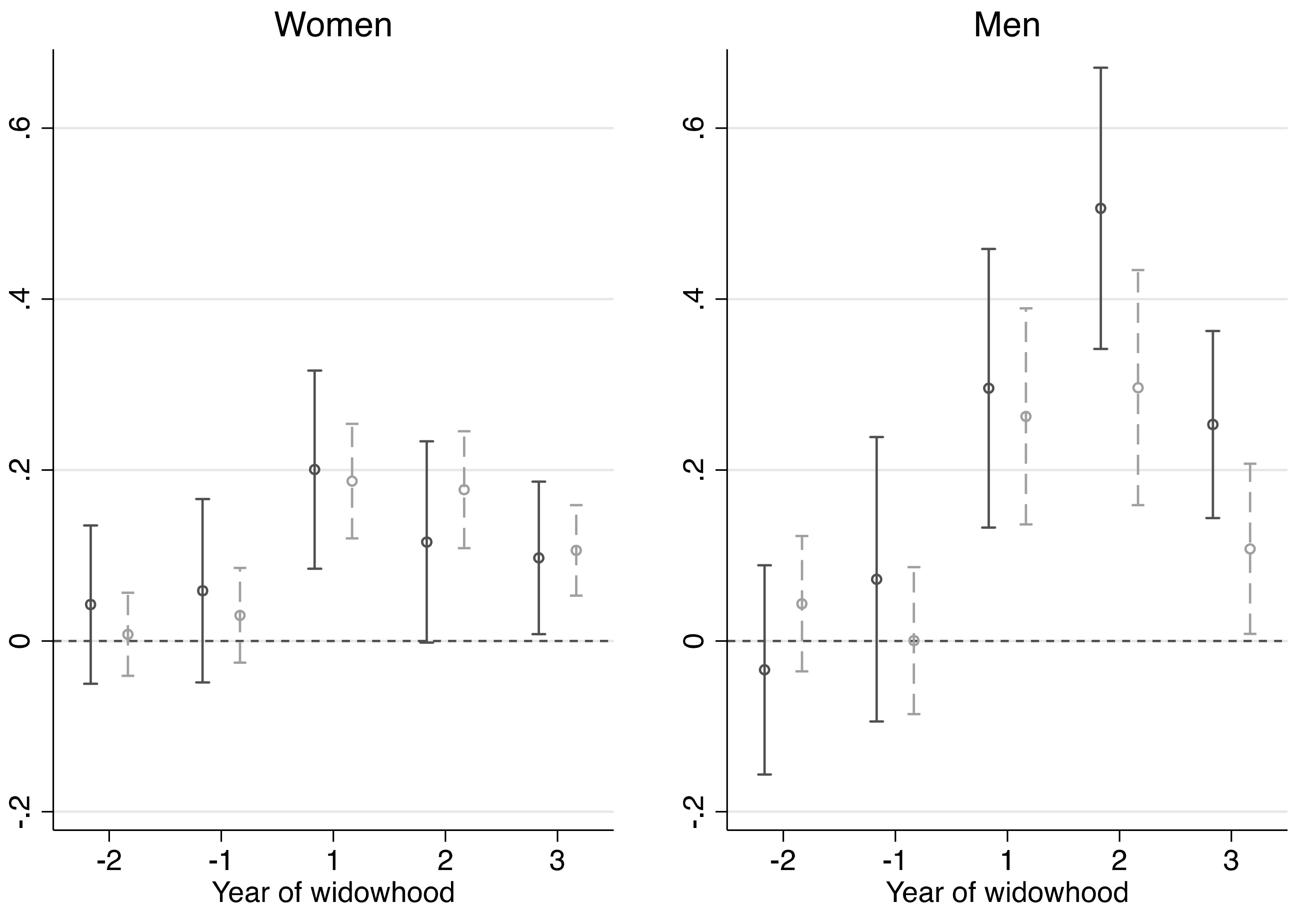

Social isolation

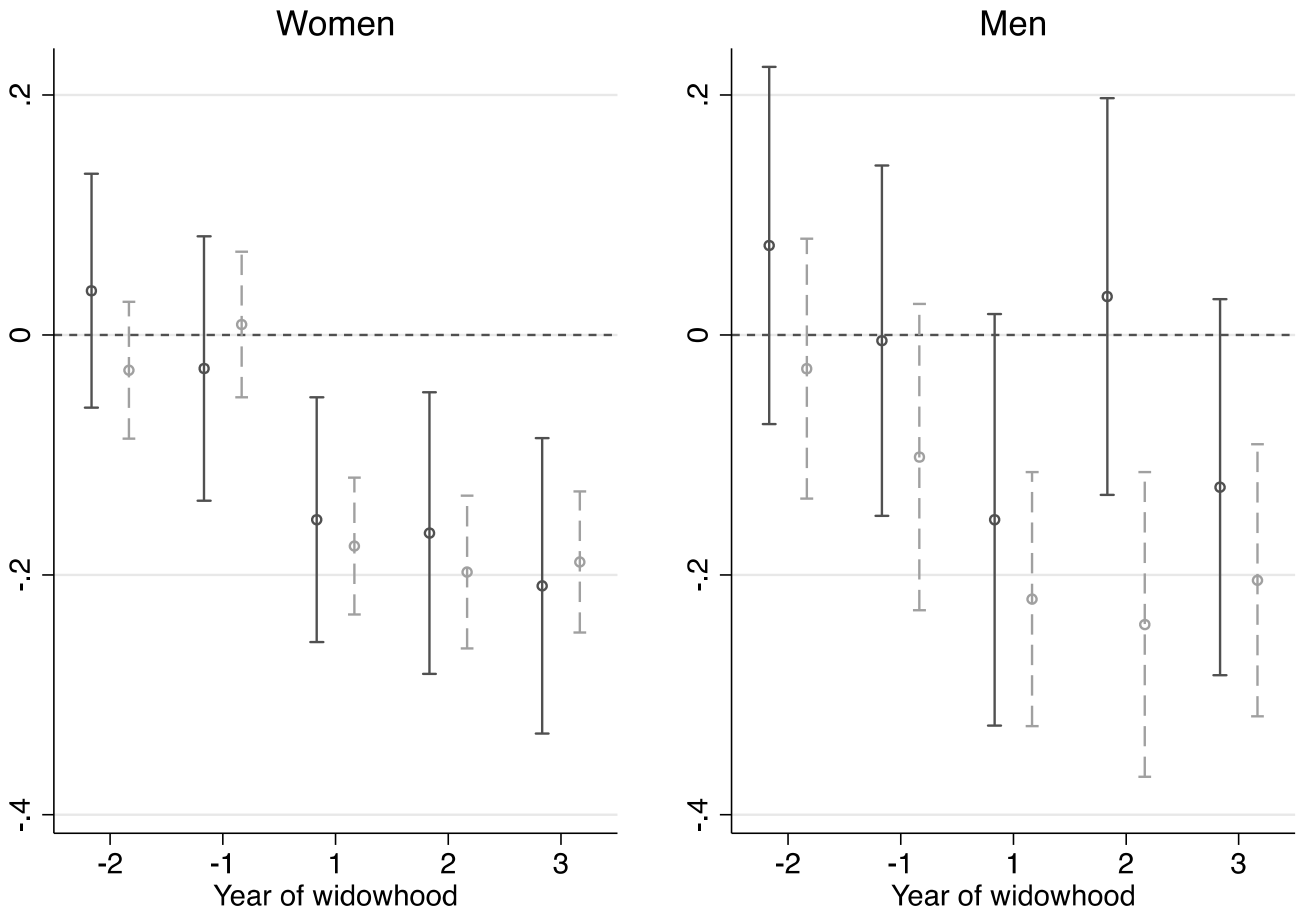

Table A1. Effects of Widowhood on Social Health from Individual Fixed-Effects Regression Models, Using Raw Rating Scores for Social Health

	Lonely (I often feel very lonely)		Social isolation (Less than weekly social get-togethers with non-household members)		Lack of social support (I don't have anyone that I can confide in)	
	Women	Men	Women	Men	Women	Men
(1) Widowhood status						
Widowed	0.707***	1.303***	-0.591***	-0.414***	-0.087	0.075
	[0.542, 0.871]	[1.026, 1.580]	[-0.705, -0.476]	[-0.613, -0.214]	[-0.224, 0.050]	[-0.136, 0.286]
(2) Year of widowhood						
- 2 years	0.178*	0.002	0.062	0.088	-0.092	-0.024
	[-0.006, 0.362]	[-0.294, 0.298]	[-0.068, 0.193]	[-0.130, 0.307]	[-0.263, 0.079]	[-0.305, 0.258]
- 1 year	0.171*	0.302*	0.083	-0.159	0.094	0.354**
	[-0.023, 0.366]	[-0.028, 0.633]	[-0.062, 0.227]	[-0.408, 0.090]	[-0.104, 0.292]	[0.041, 0.667]
+ 1 year	1.048***	1.719***	-0.491***	-0.476***	-0.107	0.189
	[0.825, 1.271]	[1.353, 2.085]	[-0.635, -0.347]	[-0.733, -0.220]	[-0.299, 0.086]	[-0.103, 0.481]

+ 2 years	0.941***	1.911***	-0.588***	-0.387***	0.059	0.119
	[0.721, 1.161]	[1.505, 2.316]	[-0.740, -0.435]	[-0.657, -0.117]	[-0.144, 0.262]	[-0.243, 0.480]
+ 3 years	0.597***	1.099***	-0.597***	-0.437***	-0.113	0.129
and after	[0.399, 0.796]	[0.776, 1.423]	[-0.745, -0.449]	[-0.693, -0.181]	[-0.285, 0.059]	[-0.136, 0.394]
Sample mean	2.493	2.360	3.526	3.748	2.309	2.492
Widowed (Obs; Indiv)	6518; 543	2337; 198	6470; 545	2332; 198	6498; 543	2341; 198
Married (Obs; Indiv)	38724; 3871	39212; 4095	38824; 3873	38755; 4107	38848; 3871	39126; 4098

Note. Figures are unstandardised B coefficient estimates and 95% confidence intervals (in square brackets). Raw ratings of the social health outcomes were used, where outcomes ranged from 1 to 7. All regressions included age, age-squared, and year dummy variables. For widowed individuals in the Panel (2) regression models, the reference period was over two years before spousal death. Obs = number of observations; Indiv = number of individuals.

*p<.10, **p<.05, ***p<.01.

Table A2. Effects of Widowhood on Social Health from Individual Fixed-Effects Regression Models, Using a Balanced Panel

	Lonely (I often feel very lonely)		Social isolation (Less than weekly social get-togethers with non-household members)		Lack of social support (I don't have anyone that I can confide in)		No formal social participation (Not a member of a club or association)	
	Women	Men	Women	Men	Women	Men	Women	Men
(1) Widowhood status								
Widowed	0.161***	0.288***	-0.174***	-0.134***	-0.020	0.001	-0.022	0.022
	[0.112, 0.209]	[0.209, 0.367]	[-0.220, -0.129]	[-0.208, -0.060]	[-0.062, 0.021]	[-0.065, 0.067]	[-0.064, 0.020]	[-0.050, 0.093]
(2) Year of widowhood								
- 2 years	-0.034	-0.062	-0.047	-0.032	-0.028	0.060	0.003	0.017
	[-0.100, 0.031]	[-0.158, 0.034]	[-0.117, 0.022]	[-0.140, 0.075]	[-0.091,0.035]	[-0.044, 0.165]	[-0.047, 0.052]	[-0.064, 0.098]
- 1 year	-0.012	-0.052	-0.025	-0.059	-0.039	0.054	0.001	0.017
	[-0.073, 0.050]	[-0.161, 0.057]	[-0.098, 0.048]	[-0.181, 0.063]	[-0.106, 0.028]	[-0.050, 0.157]	[-0.060, 0.061]	[-0.076, 0.110]
+ 1 year	0.141***	0.261***	-0.180***	-0.150***	-0.090***	0.017	-0.002	0.022
	[0.069, 0.213]	[0.142, 0.380]	[-0.255, -0.106]	[-0.263, -0.038]	[-0.154, -0.025]	[-0.093, 0.128]	[-0.063, 0.059]	[-0.079, 0.124]
+ 2 years	0.157***	0.322***	-0.194***	-0.175***	-0.029	0.091*	-0.029	-0.002
	[0.084, 0.231]	[0.194, 0.449]	[-0.266, -0.122]	[-0.303, -0.047]	[-0.097, 0.039]	[-0.013, 0.196]	[-0.097, 0.038]	[-0.100, 0.095]

+ 3 years	0.138***	0.167***	-0.220***	-0.169***	-0.009	0.009	-0.031	0.078
and after	[0.062, 0.214]	[0.059, 0.275]	[-0.294, -0.147]	[-0.277, -0.060]	[-0.079, 0.060]	[-0.099, 0.118]	[-0.099, 0.036]	[-0.040, 0.196]
Sample mean	0.163	0.141	0.412	0.476	0.155	0.172	0.493	0.500
Widowed								
(Obs; Indiv)	1368; 228	504; 84	1350; 225	498; 83	1368; 228	510; 85	1404; 234	504; 84
Married								
(Obs; Indiv)	39330; 3871	29399; 3077	39904; 3873	28888; 3090	39442; 3871	29299; 3080	39326; 3875	29302; 3091

Note. Figures are unstandardised B coefficient estimates and 95% confidence intervals (in square brackets). Dichotomised versions of the social health outcomes were used, so estimates can be interpreted as changes in the likelihood of experiencing poor social health. All regressions included age, age-squared, and year dummy variables. For widowed individuals in the Panel (2) regression models, the reference period was over two years before spousal death. Obs = number of observations; Indiv = number of individuals.

*$p<.10$, **$p<.05$, ***$p<.01$.
